# Assessing a WeChat-Based Integrative Family Intervention (WIFI) for Schizophrenia: Protocol for a Stepped-Wedge Cluster Randomized Trial

**DOI:** 10.2196/18538

**Published:** 2020-08-25

**Authors:** Yu Yu, Tongxin Li, Shijun Xi, Yilu Li, Xi Xiao, Min Yang, Xiaoping Ge, Shuiyuan Xiao, Jacob Tebes

**Affiliations:** 1 Department of Social Medicine and Health Management Xiangya School of Public Health Central South University Changsha China; 2 Division of Prevention and Community Research Department of Psychiatry Yale School of Medicine New Haven, CT United States; 3 Department of Psychiatry Changsha Psychiatric Hospital Changsha China

**Keywords:** schizophrenia, family intervention, WeChat, psychoeducation, peer support, professional support, stepped wedge

## Abstract

**Background:**

Schizophrenia is a persistent and debilitating mental illness, and its prognosis depends largely on supportive care and systematic treatment. In developing countries like China, families constitute the major caregiving force for schizophrenia and are faced with many challenges, such as lack of knowledge, skills, and resources. The approach to support family caregiving in an accessible, affordable, feasible, and cost-effective way remains unclear. The wide-spread use of WeChat provides a promising and cost-effective medium for support.

**Objective:**

We aim to present a protocol for assessing a WeChat-based integrative family intervention (WIFI) to support family caregiving for schizophrenia.

**Methods:**

We will develop a WIFI program that includes the following three core components: (1) psychoeducation (WeChat official account), (2) peer support (WeChat chat group), and (3) professional support (WeChat video chat). A rigorous stepped-wedge cluster randomized trial will be used to evaluate the implementation, effectiveness, and cost of the WIFI program. The WIFI program will be implemented in 12 communities affiliated with Changsha Psychiatric Hospital through the free medicine delivery process in the 686 Program. The 12 communities will be randomized to one of four fixed sequences every 2 months during an 8-month intervention period in four clusters of three communities each. Outcomes will be assessed for both family caregivers and people with schizophrenia. Family caregivers will be assessed for their knowledge and skills about caregiving, social support, coping, perceived stigma, caregiver burden, family functioning, positive feelings, and psychological distress. People with schizophrenia will be assessed for their symptoms, functioning, quality of life, recovery, and rehospitalization. Cost data, such as intervention costs, health care utilization costs, and costs associated with lost productivity, will be collected. Moreover, we will collect process data, including fidelity and quality of program implementation, as well as user attitude data. Treatment effects will be estimated using generalized linear maximum likelihood mixed modeling with clusters as a random effect and time as a fixed effect. Cost-effectiveness analysis will be performed from the societal perspective using incremental cost-effectiveness ratios. Qualitative analysis will use the grounded theory approach and immersion-crystallization process.

**Results:**

The study was funded in August 2018 and approved by the institutional review board on January 15, 2019. Preliminary baseline data collection was conducted in May 2019 and completed in September 2019. The WIFI program is expected to start in September 2020.

**Conclusions:**

This is the first study to assess a WeChat-based mHealth intervention to support family caregiving for schizophrenia in China. The innovative study will contribute to the development of a more cost-effective and evidence-based family management model in the community for people with schizophrenia, and the approach could potentially be integrated into national policy and adapted for use in other populations.

**Trial Registration:**

ClinicalTrials.gov NCT04393896; https://clinicaltrials.gov/ct2/show/NCT04393896.

**International Registered Report Identifier (IRRID):**

PRR1-10.2196/18538

## Introduction

### Schizophrenia and Family Caregiving

Globally, schizophrenia is a debilitating persistent psychiatric disorder affecting over 21 million people [1,2], and there is a 60% increase in premature deaths among people living with schizophrenia compared with the general population [3]. The most recent global burden of disease study in 2016 showed that schizophrenia contributes 13.4 million years of life lived with disability to the burden of disease globally [2]. The prognosis of schizophrenia depends largely on integrated mental health and social care services in community-based settings, which has been listed by the World Health Organization (WHO) as one of the four major objectives in its Mental Health Action Plan 2013-2020 [3]. Among the multiple initiatives proposed by the WHO [3], strengthening the active involvement and support of family caregivers in caring for people living with schizophrenia stands out as the most sustainable and cost-effective solution for addressing the worldwide treatment gap in resource-poor settings. Recent years have seen a global shift in the responsibility of care from the hospital setting to families [4], such that the economic value of informal family caregiving now greatly exceeds spending through formal health care systems. Recently, the Chinese government recognized the value of family caregiving by instituting the Reward Policy (described below) to support family caregivers financially [5]; however, this policy is an exception globally [6].

### The Reward Policy and Challenges

In China, there are over 7.16 million people living with schizophrenia [7], and over 90% of them live with and depend on their families for care [8]. Family caregiving often requires a range of support that extends across physical, psychological, emotional, social, and financial domains [9]. The essential roles of family caregivers in the care of schizophrenia have been increasingly recognized in China’s mental health policy. In 2016, the Chinese government instituted a Reward Policy to encourage family involvement in the care of people with serious mental illness. According to the Reward Policy, a monthly subsidy equal to the local poverty line allowance (currently at least RMB 200 or US $28.6) is paid to each family based on good management and care of the family member with serious mental illness, including schizophrenia [5,10].

Three years after its implementation, the Reward Policy has helped alleviate the financial burden for caregiving families. However, family caregivers are still faced with other challenges during the process of caregiving, such as insufficient knowledge and skills in providing appropriate care to people living with schizophrenia, social isolation due to stigma, stress from caregiving, family conflict on task-sharing, and lack of self-care due to overwhelming caregiving responsibilities [4,11,12]. As a result, family caregivers experience a considerable level of burden that not only detrimentally impacts their own health and well-being, but also leads to poor prognosis in people living with schizophrenia owing to impaired quality of care. These challenges have stimulated a focus beyond simply identifying caregiver burden to also developing effective family interventions to reduce this burden and improve care [13].

### Family Intervention Programs

To date, several family intervention programs have been developed and tested, with the following three elements identified as most promising and feasible: (1) psychoeducation for families to increase knowledge about schizophrenia and strengthen related caregiving skills; (2) peer-support for both family caregivers and people living with schizophrenia where they can share experiences and feelings, exchange information, and provide mutual emotional support; and (3) professional support to family caregivers that troubleshoots specific problems and provides private targeted guidance to address specific needs [13-18]. Thus far, integrative application of all three intervention components has been limited in China owing to their low accessibility and high cost, and evidence on their combined use and effectiveness to support family caregivers and people living with schizophrenia is lacking. An innovative, affordable, and cost-effective platform that integrates all three of these intervention components thus represents a pressing need in the scientific literature and for the national health care policy.

### WeChat Use in China

WeChat is the most common social media platform in China, with over 1 billion monthly active users of all ages [19]. About 93% of urban users log into WeChat every day [20]. WeChat features diverse platforms, such as moments, chat group, and WeChat official account (WOA), and boasts of multiple powerful functions including voice and text messaging, voice and video calls, photo sharing, payment, and games. Owing to its wide range of platforms and functions, WeChat has been dubbed China’s “app for everything” and has been characterized as “4A” (anybody, anytime, anywhere, and anything) [21,22]. The seamless integration of WeChat into every aspect of human life makes it a promising and cost-effective medium for health intervention delivery. A growing number of WeChat-based health intervention programs have been developed for patients with various health conditions, with robust evidence showing their acceptability, feasibility, and efficacy [23-27]. Specifically, WeChat-based health interventions have been found to cost less, improve treatment adherence, have fewer complications, increase rates of follow-up, require less intervention time, and improve patient satisfaction [23-27]. Thus, we hypothesize that a WeChat-based Integrative Family Intervention (WIFI) program that includes the three elements noted above (psychoeducation, peer support, and professional support) will be an accessible and cost-effective approach to improve the outcomes of both people living with schizophrenia and family caregivers.

### Theoretical Framework for the Proposed Study

**Figure 1:**
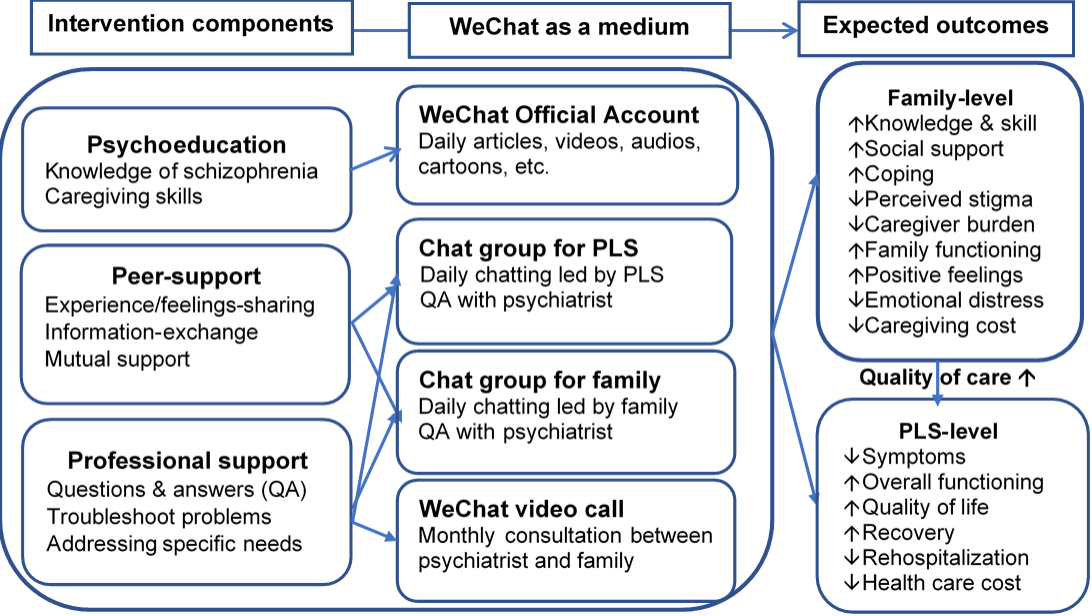
Theoretical framework of the proposed study. PLS: people living with schizophrenia.

The theoretical mechanisms underlying the proposed study are psychoeducation, peer support, and professional support (Figure 1). There is empirical evidence that each of these mechanisms promotes the expected outcomes examined. Psychoeducation is central to the proposed study because it directly increases knowledge about schizophrenia and caregiving and indirectly works through the actions of peers and professionals in the provision of support. More specifically, there is considerable evidence that psychoeducation has been widely employed with caregivers and with people living with schizophrenia to yield a range of positive effects. For example, with caregivers, psychoeducation has been shown to increase knowledge and skills [28], improve social support [29] and coping [30,31], improve family functioning [32], decrease stigma [28] as well as family burden [33-35], promote positive feelings and decrease emotional distress [34-36], and reduce the cost of care [37]. Furthermore, for people living with schizophrenia, psychoeducation has been shown to decrease symptoms, improve functioning, enhance quality of life, increase recovery, decrease hospitalizations, and reduce health care costs [15,38-40]. Importantly, psychoeducation also yields positive effects for caregivers and people living with schizophrenia when delivered through peer support and professional support [18,41-43].

Consistent with the literature, three components are included in the WIFI program to provide education and support to families (psychoeducation, peer support, and professional support) (Figure 1). WeChat will provide access for caregivers and people living with schizophrenia to each of these intervention components. Psychoeducation and support will increase knowledge and skills, as well as social support (peer and professional) and coping to reduce perceived stigma and caregiver burden. In addition, these components are expected to enhance family functioning and positive feelings, such that emotional distress will be reduced. Finally, these effects are hypothesized to reduce caregiving costs. For people living with schizophrenia, these components are expected to enhance the overall quality of care in the community, which is hypothesized to reduce symptoms in people living with schizophrenia and enhance functioning, increase quality of life and recovery, and decrease rehospitalization, thus reducing overall health care costs.

This paper describes the protocol of a study designed to assess the impact of a WIFI program fully aligned with the Reward Policy for families caring for people living with schizophrenia compared with the Reward Policy alone. The specific aims are as follows: (1) compare the effects of the WIFI program plus the Reward Policy with the Reward Policy alone on caregiving and the health outcomes of family caregivers and people living with schizophrenia, such as knowledge and skills, social support and coping, burden, family functioning, positive feelings, and psychological distress of caregivers, as well as symptoms, functioning, and recovery of people living with schizophrenia; (2) compare the total cost of the WIFI program plus the Reward Policy with the Reward Policy alone, including the program itself, health care utilization of people living with schizophrenia and family caregivers, and production loss of family caregivers; and (3) conduct a process evaluation of the WIFI program to assess fidelity and quality of program implementation, as well as user attitudes toward the program. To simultaneously assess intervention effectiveness and implementation strategies using mixed methods in “real-life” health care settings, we used a stepped-wedge cluster randomized trial (SWCRT) design [44].

## Methods

### Setting

The study will be conducted at Changsha Psychiatric Hospital (also named The Ninth Hospital of Changsha). Established in 1952 and affiliated to Changsha Civil Affairs Bureau, Changsha Psychiatric Hospital has the responsibility of prevention, treatment, and rehabilitation for all residents with mental illnesses in Changsha City. The hospital not only provides out-patient and in-patient health care, but also extends its services to community-based mental health care for its 12 affiliated communities, including the “686 Program” and Reward Policy implementation. The “686 Program” is China’s largest demonstration project in mental health service aimed at integrating hospital and community services for serious mental illnesses, with the following services provided mainly through community health workers: patient registration and initial assessment, free medication and regular follow-up in the community, management of community emergencies, and free emergency hospitalization [45-47]. In Changsha Psychiatric Hospital, a medical team involving three psychiatrists and four nurses is responsible for the “686 Program,” and the members circulate around the 12 communities each month to delivery free medicines to over 1000 registered clients who they know very well after long-term visits. The Reward Policy is a newly issued policy to encourage family care of people with serious mental illness, with RMB 200 (US $28.6) per month currently offered to each family registered under the 686 Program by Changsha Psychiatric Hospital.

### Design

This study uses a pragmatic stepped-wedge design [44] to evaluate both the effectiveness and implementation strategy of the WIFI program. The CONSORT checklist is presented in Multimedia Appendix 1, and the SPIRIT checklist is presented in Multimedia Appendix 2. We will conduct a multicenter prospective controlled trial, using a stepped-wedge design, comparing the WIFI program integrated into the Reward Policy (intervention group) and the Reward Policy alone (control group) in family caregiving among people living with schizophrenia.

In a SWCRT, all clusters are randomly and sequentially crossed over from control to intervention over a number of time periods [48]. All clusters serve as a control group at the beginning of the study and end up in the intervention group at the end of the study. Compared with traditional randomized controlled trials and parallel cluster studies, a SWCRT enjoys unique ethical benefits since all clusters will ultimately receive the assumingly beneficial intervention at the end of the study [49]. In addition, a SWCRT enables analyses of any temporal effects of the intervention since each cluster acts as its own control and also allows for estimation of both between- and within-cluster effects of the intervention owing to repeated measurements [49]. As a result, a SWCRT design achieves greater statistical power with smaller sample sizes and is more cost-effective than parallel group designs.

**Figure 2:**
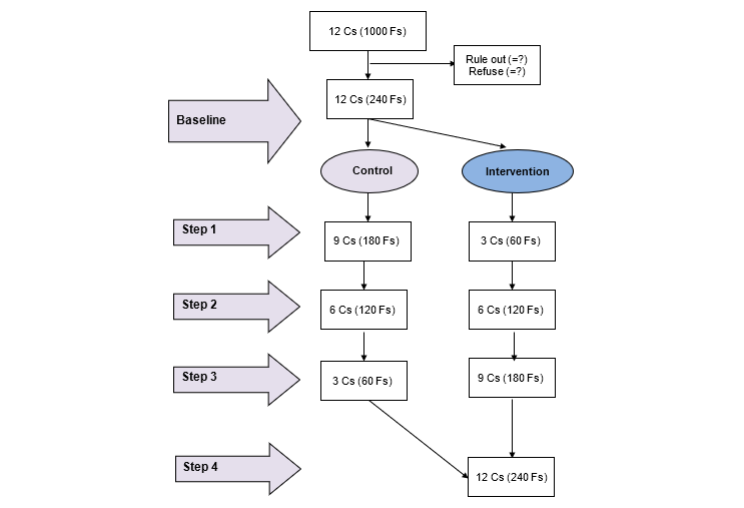
Flowchart for participant recruitment and allocation. C: community; F: family.

In the proposed study, a WIFI program will be implemented sequentially across an 8-month intervention period in the 12 communities by Changsha Psychiatric Hospital. A total of 20 families will be recruited from each community, leading to a total sample of 240 families. Allocation will be determined by an external statistician using a computer-generated random number sequence. Each number will be secretly and securely stored in a sealed envelope by the external statistician until the intervention starts. The first author (YY) will generate the allocation sequence, the medical team will work on enrolling participants, and the second author (TXL) will assign participants to interventions. The 12 communities will be randomized into four groups according to geographic distance and number of people living with schizophrenia to reduce the risk of contamination and group size inequality. After allocation, each group will be randomized to one of four fixed sequences every 2 months during an 8-month intervention period (Table 1 and Figure 2). All communities will receive the usual financial benefit of the Reward Policy as the control condition before the intervention, and then, successively and in random order, will cross over to the WIFI program at 2-month intervals until the study ends.

**Table 1:** Design of the four-stage stepped-wedge cluster randomized trial.

Cluster community (CM) group^a^	Assessment^b^				
	Baseline (M_1-2_)^c^	Step 1 (M_3-4_)	Step 2 (M_5-6_)	Step 3 (M_7-8_)	Step 4 (M_9-10_)
CM_1-3_	C^d^	WIFI^e^	WIFI	WIFI	WIFI
CM_4-6_	C	C	WIFI	WIFI	WIFI
CM_7-9_	C	C	C	WIFI	WIFI
CM_10-12_	C	C	C	C	WIFI

^a^Number of clusters=12; number of groups=4; number of clusters per group=3.

^b^Step length=2 months; number of participants per step=20.

^c^M: month.

^d^C: control (Reward Policy alone).

^e^WIFI: WeChat-based Integrative Family Intervention.

### Participants and Recruitment

Recruitment is estimated to start in September 2020. The study aims at recruiting 240 families of people living with schizophrenia from 12 communities affiliated to Changsha Psychiatric Hospital through the “686 Program.” Within each community, 20 eligible people living with schizophrenia will be randomly selected from the registry name list by a statistician, leading to a sampling frame of 240 families of people living with schizophrenia. Each family will be approached and invited to participate in the study during the monthly medicine delivery by the medical team from Changsha Psychiatric Hospital. We do not require both a person living with schizophrenia and a family caregiver to be recruited into the study at the same time. The entire family may benefit from the intervention as long as there is one member from the family participating*.* The medical team has been providing mental health services, including free antipsychotic medicine delivery, in the communities for a long time and thus knows very well about each family having a person living with schizophrenia, which greatly facilitates participant recruitment and retention. Detailed information about the research will be provided both orally and in written format to interested families by the medical team. All families will be fully informed of the study risks and benefits, and their right to drop out of the study at any time (Multimedia Appendix 3). Families agreeing to participate in the study will be invited to scan a WeChat barcode of the research program, so that they can be allocated to receive the intervention in the future. Our research team will include three psychiatrists who will complete clinical assessments of symptoms and functioning of the people living with schizophrenia through WeChat video chat. In addition, 10 postgraduate students will assist people living with schizophrenia and their caregivers to complete online questionnaires through WeChat. The research team will receive extensive training for both the intervention and evaluation to ensure quality and consistency.

Participants of the study will include both people living with schizophrenia and their family members. The inclusion criteria for participating people living with schizophrenia are as follows: (1) registration in the “686 Program;” 2) fulfilling the Chinese Classification of Mental Disorders-3 (CCMD-3) or the International Classification of Diseases-10 (ICD-10) criteria for schizophrenia; (3) age 18 years or older; (4) living with at least one family member; and (5) ability to use a smartphone and WeChat to read and communicate. The inclusion criteria for participating family members are as follows: (1) registration in the Reward Policy and receiving a subsidy for family care; (2) living with a person having schizophrenia for at least the past 2 years; (3) age 18 years or older; 4) involvement with caregiving activities of people living with schizophrenia; (5) ability to use a smartphone and WeChat to read and communicate; and (6) at least one family member having a smartphone with the WeChat app installed.

### Blinding

People living with schizophrenia, family caregivers, medical team members, and researchers cannot be blinded to the allocated treatment. The program team conducting the intervention will not be involved in assessing any of the outcomes. The data analyses by researchers will be blinded.

### Intervention

Participants in the control group will receive the usual financial benefits of the Reward Policy and receive payments from Changsha Psychiatric Hospital. However, they will not have access to the WIFI program since they cannot scan the WeChat barcode for the research.

Participants in the intervention group will receive the usual financial benefits of the Reward Policy as well as the WIFI program that will include the following three key components: psychoeducation through WOA publications, peer-support through a WeChat chat group, and professional support through WeChat private chat and video calls (Table 2).

**Table 2:** Content of the WeChat-based integrative family intervention program.

Component	Format	Frequency	Leader	Possible content/topics
Psychoeducation	WeChat official account publications	Weekly	Psychiatrists and researchers	What is schizophrenia? What causes schizophrenia? How is schizophrenia treated? What can be done to promote recovery in schizophrenia? What are the early signs of relapse? What support do families need? How can feelings of stigma be addressed?
Peer support	WeChat chat group of people living with schizophrenia	Daily	People living with schizophrenia volunteers	Introduce self and tell your story; identify a specific problem encountered to discuss with the group; discuss skills and techniques used to cope with challenging situations; share feelings and resources; organize offline activities for support and stress reduction, such as hiking, dinner, and group meetings.
Peer support	WeChat chat group of caregivers	Daily	Caregiver volunteers	Introduce self and tell your story; identify a specific problem encountered to discuss with the group; discuss skills and techniques used to cope with challenging situations; share feelings and resources; organize offline activities for support and stress reduction, such as hiking, dinner, and group meetings.
Professional support	Private WeChat chat and video call	Monthly	Psychiatrists	Evaluate symptoms and function in people living with schizophrenia; update on medication and treatment; troubleshoot specific problems; provide consultation, guidance, assistance, etc.

### Contamination

Since randomization is performed at the community level, using the stepped-wedge design, the risk of contamination between the control and intervention groups is very low. Moreover, since the intervention is delivered through WeChat and each participant scans the special WeChat barcode of the research program to obtain access to the WIFI program, it is unlikely that participants in the control group will receive the intervention during the control stage. Even if participants in the control group learn about the WeChat account of the research program, they will not be able to add it because the research team will recognize each participant and decline any request from the control group until receiving the allotted sequence to join the intervention. Thus, intervention contamination will be avoided.

### Outcomes

#### Effect Measures

The effect of the intervention will be assessed at the individual level for both family caregivers and people living with schizophrenia.

For family caregivers, the outcomes will include knowledge and skills about caregiving (Knowledge and Skill of Caregiving for Schizophrenia, self-developed), social support (Multidimensional Scale of Perceived Social Support) [50], coping (Simplified Coping Style Questionnaire) [51], perceived stigma (Perceived Devaluation and Discrimination Scale) [52], caregiver burden (Zarit Burden Interview [ZBI]) [53], family functioning (Family Adaptation, Partnership, Growth, Affection and Resolve Index scale) [54,55], positive feelings (Caregiving Rewarding Feelings) [56], perceived stress (Perceived Stress Scale) [57], depression (Patient Health Questionnaire-9 [PHQ-9]) [58], and anxiety (Generalized Anxiety Disorder Scale-7 [GAD-7]) [59].

For people living with schizophrenia, the outcomes will include clinical symptoms (Brief Psychiatric Rating Scale [BPRS]) [60] and overall functioning (Global Assessment of Functioning [GAF]) [61], which will both be rated by psychiatrists. Other outcomes will include self-reported quality of life (World Health Organization Quality of Life Brief Scale) [62], recovery (Recovery Assessment Scale [RAS]) [63], rehospitalization, depression (PHQ-9) [58], and anxiety (GAD-7) [59].

#### Potential Confounding Factors

At baseline, we will also collect information about potential confounding factors by adjusting for the following: (1) sociodemographic data, such as age, gender, education, and occupation; (2) clinical data, such as diagnosis type of schizophrenia, length of illness, and length of caregiving; and (3) WeChat use intensity as assessed by the WeChat Use Intensity Questionnaire [64,65].

#### Cost Measures

Costs will be measured from a societal perspective and consist of at least the following three levels: (1) costs of the intervention, (2) health care utilization costs, and (3) costs associated with lost productivity. All of the costs will be converted to that for the year 2019 using consumer price indices.

The intervention costs pertain to implementation and operation of the WIFI program. A bottom-up approach will be used to assess the intervention costs, which may include but are not limited to (1) training of psychiatrists, researchers, and other project team members and (2) the WeChat intervention (WOA fee, administrator time, consultation fee of the psychiatrists, etc).

The health care utilization costs pertain to medical care for both people living with schizophrenia and family caregivers. A monthly cost diary will be used to retrospectively track medical expenses incurred by people living with schizophrenia and family caregivers, which may include but are not limited to (1) visits to health care professionals in primary or secondary care; (2) hospitalization; (3) visits to alternative medicine therapists; (4) medication; and (5) other nonmedical expenses associated with medical care, such as transportation, food, and lodging. Health care utilization costs will be estimated by China guideline prices that are supplemented by population-based estimates in the literature.

The costs associated with productivity loss will be assessed at both the people living with schizophrenia and caregiver levels. For both people living with schizophrenia and caregivers, costs include absenteeism due to sick leave, which will be assessed by monthly sick leave calendars. The human capital approach will be used to calculate the costs of losses to production due to sickness or caregiving (net number of days on leave during follow-up multiplied by the daily wage of the worker if employed or an equivalent value if unemployed).

#### Process Measures

A process evaluation will be conducted to evaluate the implementation process of the intervention to understand potential factors related to implementation that may be associated with observed outcomes. The evaluation includes fidelity and quality of WIFI implementation, as well as users’ attitudes toward the program, which will be evaluated separately for people living with schizophrenia, their family members, and psychiatrists. After completion of the intervention in each randomized community, both quantitative and qualitative process data will be collected from survey samples of people living with schizophrenia and family members to assess their awareness of and responsiveness to the WIFI program.

Quantitative data will be directly collected through the WeChat backstage management system and include information about families’ use of and engagement with the WIFI program. For psychoeducation data, we will collect information on views, downloads, and shares of WOA publications. For peer-support data, we will collect information on chatting topics, number of messages sent, and active users of the WeChat chat group through chat records. For professional support, we will collect information on help-seeking behaviors of families, number of consultations, problems addressed by psychiatrists, etc.

Qualitative information will be collected through online one-to-one video interviews using the video chat function of WeChat. The technology acceptance model will be used to explore perceived usefulness and perceived ease of use of the WIFI program among people living with schizophrenia, family members, and psychiatrists at the end of the intervention [66-69]. People living with schizophrenia and family members will be asked about their feelings and experiences with the WIFI program, such as attitudes, beliefs, and feedback about the program. Psychiatrists will be asked about their exposure to and experiences with each element of the WIFI program in order to find both facilitators and barriers of program implementation at the provider level. All this information will help the research team gain insights into the feasibility and replicability of the program.

### Data Collection and Management

Data are collected from people living with schizophrenia, their family members, and psychiatrists at baseline (months 1-2) and at 4, 6, 8, and 10 months. A pilot study with face-to-face interviews was recently completed with 400 families of people living with schizophrenia prior to the formal WIFI program to test all measures and collect baseline data. For this data collection, all participants will be invited through WeChat to complete questionnaires through an online survey known as Sojump [70]. Sojump provides a series of services including questionnaire design and distribution, data collection, and analysis. In addition, all qualitative information (on process measures) will be collected by online one-to-one interviews through WeChat video chat. Each family will be reimbursed with money for participation each time, which will depend on the completion of their relevant questionnaires (about 20 minutes for the people living with schizophrenia and 45 minutes for the caregivers). The reimbursement will increase by 25% for each successive assessment to reflect participants’ ongoing study commitment. Specifically, participants will receive RMB 35 (US $5) for the baseline measurement, followed by RMB 44 (US $6.25), RMB 55 (US $7.81), RMB 69 (US $9.77), and RMB 86 (US $12.21) for the subsequent assessments. A family will be reimbursed with a total of RMB 289 (about US $41) for completion of all five assessments. Payment will be sent directly to one designated family member through the WeChat money transfer function. The double entry method will be adopted to input data, with the range for data values preset to avoid any wrong input. All data will be safely stored on a disk and managed by a special data specialist. Table 3 provides an overview of all outcome measures and assessment instruments that will be used in this trial.

**Table 3:** Assessment of study outcomes.

Outcome measures		Assessment				
		M_1-2_^a^	M_3-4_	M_5-6_	M_7-8_	M_9-10_
**Caregivers**						
	Knowledge and skill (Knowledge and Skill of Caregiving for Schizophrenia)	Yes	Yes	Yes	Yes	Yes
	Social support (Multidimensional Scale of Perceived Social Support)	Yes	Yes	Yes	Yes	Yes
	Coping (Simplified Coping Style Questionnaire)	Yes	Yes	Yes	Yes	Yes
	Perceived stigma (Perceived Devaluation and Discrimination Scale)	Yes	Yes	Yes	Yes	Yes
	Caregiver burden (Zarit Burden Interview)	Yes	Yes	Yes	Yes	Yes
	Family functioning (Family Adaptation, Partnership, Growth, Affection and Resolve Index scale)	Yes	Yes	Yes	Yes	Yes
	Positive feelings (Caregiving Rewarding Feelings)	Yes	Yes	Yes	Yes	Yes
	Perceived stress (Perceived Stress Scale)	Yes	Yes	Yes	Yes	Yes
	Depression (Patient Health Questionnaire-9)	Yes	Yes	Yes	Yes	Yes
	Anxiety (Generalized Anxiety Disorder Scale-7)	Yes	Yes	Yes	Yes	Yes
**People living with schizophrenia**						
	Symptoms (Brief Psychiatric Rating Scale)	Yes	Yes	Yes	Yes	Yes
	Functioning (Global Assessment of Functioning)	Yes	Yes	Yes	Yes	Yes
	Quality of life (World Health Organization Quality of Life Brief Scale)	Yes	Yes	Yes	Yes	Yes
	Recovery (Recovery Assessment Scale)	Yes	Yes	Yes	Yes	Yes
	Rehospitalization	Yes	Yes	Yes	Yes	Yes
	Depression (Patient Health Questionnaire-9)	Yes	Yes	Yes	Yes	Yes
	Anxiety (Generalized Anxiety Disorder Scale-7)	Yes	Yes	Yes	Yes	Yes
**Potential confounding factors**						
	Social demographic variables	Yes	No	No	No	No
	Clinical variables	Yes	No	No	No	No
	WeChat use intensity (WeChat Use Intensity Questionnaire)	Yes	No	No	No	No
**Cost**						
	WIFI program (bottom-up approach)	Yes	Yes	Yes	Yes	Yes
	Health care utilization (cost dairy)	Yes	Yes	Yes	Yes	Yes
	Productivity loss (sick leave calendar)	Yes	Yes	Yes	Yes	Yes
**Process**						
	Fidelity (quantitative)	No	No	No	No	Yes
	Quality (quantitative)	No	No	No	No	Yes
	Attitudes (qualitative)	No	No	No	No	Yes

^a^M: month.

### Statistical Analysis

We will use mixed-methods analysis for both qualitative and quantitative data collected during each step of the WIFI program. For qualitative data, a grounded theory approach [71] and immersion-crystallization process [72] will be used to assess process implementation and gain deep insights into the feasibility and replicability of the WIFI program. For quantitative data, descriptive analysis will be conducted to describe the characteristics of the participants during the control and intervention periods. Continuous variables will be described by mean (SD) or median (IQR) depending on the shape of the distribution. Categorical variables will be described by number and percentage in each category. For two-group comparisons, the Student *t* test or nonparametric test will be conducted for continuous variables, while the chi-square test or Fisher exact test will be conducted for categorical variables. Multiple imputations will be adopted to deal with missing values.

Treatment effects (WIFI vs control) will be estimated using generalized linear maximum modeling with clusters as a random effect and time as a fixed effect. All of the available measurements (2, 4, 6, 8, and 10 months) will be used, with the baseline values of each outcome as a covariate. This analysis will take into account the within-cluster and between-cluster correlations, as well as any evolution of the intervention effect over time. Statistical analyses will be performed at the individual level and according to the intention-to-treat principle, which will be compared to per-protocol analyses. Additionally, extra costs of the WIFI program will be evaluated.

### Power Analyses

To illustrate the power for analyses of both caregivers and people living with schizophrenia, we can use a baseline ZBI score of 45 for caregivers and a baseline GAF score of 42 for people living with schizophrenia based on previous studies as well as a baseline study conducted by the research team with caregivers and people living with schizophrenia in Hunan province. For a SWCRT with 12 communities over five time periods or steps (baseline and four intervention steps), assuming an intracluster correlation of 0.05, 90% power at a 5% significance level, and 20% dropout ratio, a sample size of 235 is needed to detect a clinically important decrease in caregiver burden as assessed by a decrease in the ZBI score from a baseline of 45 to 30 [50]. Similarly, for people living with schizophrenia, a sample size of 210 is needed to observe an increase in the GAF score from a baseline of 42 to 52, assuming α=.05, β=.15, and 20% attrition. Based on the above calculations, we decided on a sample size of 20 families per community (240 families in total), which will be sufficient to detect expected improvements in both caregiver burden and functioning of people living with schizophrenia.

### Cost-Effectiveness Analyses

A cost-effectiveness analysis will be performed from the societal perspective according to the intention-to-treat approach, with missing data imputed using multiple imputations [73]. CIs (95%) will be obtained by bias corrected and accelerated bootstrapping. The incremental cost effectiveness ratios will be calculated by dividing the differences in mean total costs between both groups by the difference in mean effects between both groups (eg, ZBI score of caregivers and BPRS and GAF scores of people living with schizophrenia). The incremental cost utility ratio will be calculated by dividing the incremental costs by the difference in the quality-adjusted life years assessed with the EuroQoL five-dimensional instrument (EQ-5D) between both treatment groups. These ratios will be graphically presented in a cost-effectiveness plane [74].

### Data Monitoring and Evaluation

In order to ensure the smooth progress of the intervention and protect participants’ welfare, a data monitoring committee (DMC) will be established. The DMC will involve administrative staff from both the hospital and its 12 affiliated community health centers, who are independent from the sponsor and have no competing interests. The main responsibility is to monitor the study progress and deal with any adverse event during the study process, which will be reported by the medical team to the principal investigator (YY) and then to the DMC. Every month, the DMC will hold a study meeting to summarize study progress, troubleshoot problems, and deal with any adverse events during the study. The DMC also has the right to terminate the intervention when it deems that the intervention is a large risk to participants.

### Ethical Consideration

The study protocol was reviewed and approved by the Institutional Review Board (IRB) of Xiangya School of Public Health Central South University (approval number: XYGW-2019-029). All procedures are in accordance with the Declaration of Helsinki. The WIFI program has been registered as a clinical trial (NCT04393896). Any modifications (eg, changes to eligibility criteria, outcomes, and analyses) to the study protocol will be reported to relevant parties (eg, investigators, IRB, trial participants, trial registries, journals, and regulators) immediately. The medical team will approach participants to fully inform them of the study aims and contents and acquire written informed consent from them before recruitment. The medical team will recommend the WIFI program to all people living with schizophrenia and their family members who come for free medicines, without any biased selection based on personal preference. Participation or refusal will not affect medicine acquisition and subsequent medical or nursing care. In order to protect participant confidentiality, personal information of participants will be collected only once during baseline data collection and then deidentified with newly generated numbers (instead of name, age, etc) after the trial. The deidentified data will only be shared or reported aggregately instead of individually. All data will be securely and secretly stored on a disk and managed by a special data specialist. Only the principal investigator and the research team will have access to the final trail data sets.

### Knowledge Dissemination

Trial results will be communicated to participants, health care professionals, the public, and other relevant groups through papers published in peer-reviewed journals (four to six international and two to four national), a PhD thesis, a master’s thesis, presentation at one national and one international conference, lectures targeted at people living with schizophrenia and family members in psychiatric hospitals, posters and pamphlets in community health centers, and popular science articles on WOA. Authorship will follow the ICMJE recommendation for authorship based on the criteria of substantial contributions to the conception or design of the work; acquisition, analysis, or interpretation of data for the work; drafting the work or revising it critically for important intellectual content; and final approval of the version to be published. The full protocol, participant-level data set, and statistical code are available from the first author (YY) and corresponding author (SYX) on reasonable request.

## Results

The study was funded in August 2018 and was approved by the IRB on January 15, 2019. Preliminary baseline data collection was conducted in May 2019 and completed in September 2019. The WIFI program is expected to start in September 2020.

## Discussion

Using a SWCRT design, the proposed study will develop a WIFI program fully aligned with the Reward Policy and test its effectiveness. Expected results will include the following: (1) significant improvement in outcomes for both people living with schizophrenia and their family members owing to WIFI program participation; (2) stronger impact for WIFI combined with the Reward Policy than the Reward Policy alone; and (3) development of a cost-effective replicable family management model for schizophrenia that can be integrated into the current national Reward Policy.

The study has some unique advantages and innovations. First, the WIFI program recruits the whole family of people living with schizophrenia as the intervention target, which may produce more far-reaching positive effects than interventions targeted at people living with schizophrenia alone or caregivers alone. In Asian countries like China, family cohesion and harmony are the core of the family-oriented culture. Interventions targeted at the family not only directly improve the well-being of each member, but also improve the family dynamic, which, in turn, will promote each member’s well-being. Second, the WIFI program is based on the most widely used social media platform in China (WeChat), which is accessible, affordable, feasible, and cost-effective. Compared with traditional on-site interventions, the WeChat-based intervention provides both synchronous and asynchronous communication that can serve a broad range of respondents who would otherwise not be recruited owing to time restraints and geographical constraints. Third, the WIFI program provides the most comprehensive intervention by integrating all three key components of family intervention that have been internationally recognized (psychoeducation, peer support, and private/professional support). Each component has its unique effect in improving the health outcome of people living with schizophrenia and their family members, and the components compensate each other to maximize the benefits to the family. Fourth, the stepped-wedge design has ethical advantages by ensuring all participants receive the intervention, as well as statistical advantages by generating more sound and robust scientific evidence than a traditional randomized cluster trial. Fifth, the WIFI program involves a medical team with both clinical psychiatrists and psychiatric nurses who work as both intervention implementers and data collectors. The medical team has a long close relationship with the community and is well accepted by people living with schizophrenia and their families, which can greatly increase the participant recruitment rate. In addition, the medical team knows about each person living with schizophrenia and can make more accurate assessments regarding the symptoms and functions of people living with schizophrenia, which can further increase the reliability and validity of the WIFI program.

One concern about this study is the potential attrition of participating people living with schizophrenia and their family members, which is very common in longitudinal intervention studies, especially those involving online interventions. Since all participants are recruited through the monthly medicine delivery process of the “686 Program” that has been running successfully for many years and the people living with schizophrenia are known to the medical team, we believe this stable and long-term community connection will increase program retention and reduce study attrition. Moreover, the use of the most widely accepted social media platform in China, WeChat, which is embedded in many aspects of daily life, is likely to reduce program and study attrition. Nevertheless, we account for attrition by estimating a 20% attrition rate in our study sample. In addition, the reimbursements for participants will increase by 25% for each successive assessment through the conclusion of the study to reflect participants’ ongoing commitment.

Another concern is the potential risk of privacy violation with the use of the WeChat platform as a means to deliver the intervention, especially the peer-support group through the WeChat group chat. It is likely that some personal information and chat records of participants may be disclosed intentionally or unintentionally by other participants. For each peer-support WeChat group, we will appoint a research team member to monitor participant interactions and flag privacy issues that emerge for group members. Regarding information and data collected through WeChat, we will store the data in an encrypted file managed by a member of the research team.

In conclusion, this innovative study will contribute to the development of a more cost-effective and evidence-based family management model in the community for people living with schizophrenia. The proposed study is among the first to develop and test a WeChat-based mHealth intervention to support family caregiving for schizophrenia in China. If found to be effective, the intervention could potentially be integrated into the current national policy to support family caregiving. The intervention could also be adapted for use in other populations having a persistent and disabling condition.

## Appendix

Multimedia Appendix 1 CONSORT checklist.

Multimedia Appendix 2 SPIRIT checklist.

Multimedia Appendix 3 Informed consent form.

Multimedia Appendix 4 Funding letter.

## Abbreviations

BPRS: Brief Psychiatric Rating Scale
CCMD-3: Chinese Classification of Mental Disorders-3
DMC: data monitoring committee
EQ-5D: EuroQoL five-dimensional instrument
GAD-7: Generalized Anxiety Disorder Scale-7
GAF: Global Assessment of Functioning
ICD-10: International Classification of Diseases-10
IRB: Institutional Review Board
PHQ-9: Patient Health Questionnaire-9
RAS: Recovery Assessment Scale
SWCRT: stepped-wedge cluster randomized trial
WHO: World Health Organization
WIFI: WeChat-based Integrative Family Intervention
WOA: WeChat official account
ZBI: Zarit Burden Interview
