# Supplementary material for: Assessing a WeChat-Based Integrative Family Intervention (WIFI) for Schizophrenia: Protocol for a Stepped-Wedge Cluster Randomized Trial
Source: JMIR Res Protoc. 2020 Aug 25;9(8):e18538. doi: 10.2196/18538 (PMC7479588; doi:10.2196/18538)
Supplement: Multimedia Appendix 3 [file resprot_v9i8e18538_app3.docx]

**知情同意书**

**尊敬的受访者：**

我们邀请您参加由中南大学与长沙市精神病院合作开展的《通过微信对精神分裂症家庭进行综合干预及评估：一项阶梯干预试验研究》。本研究已经得到中南大学公共卫生学院伦理委员会的审查和批准。本文涵盖的部分内容由法规要求而定，并且为了保护参与研究的参加者的权益，本文经伦理委员会审核并同意。

本研究旨在通过**微信平台**为精神分裂症患者及家属提供**在线支持和帮助**，来减轻照料负担，提升照料质量，促进患者康复，促进患者和家庭成员的身心健康。本干预分为三个核心部分：基于微信公众号的**心理教育**，基于患者和家属微信群的**同伴支持**，基于微信语音或视频聊天的**精神科医生单独家访**。本项目持续一年，分四个阶段进行，会有四次基于问卷星的干预后问卷调查，部分参与者还会接受一次基于微信视频聊天的定性访谈，了解您对本项目的体验和感受以及建议。如果您同意参与这项研究，您将**免费获得**以上所有**干预和服务**内容，同时也需要完成**四次问卷调查评估**。您对微信干预项目公众号的浏览记录，您在同伴支持群里的聊天记录，以及您与精神科医生的家访聊天内容都会被研究者获取并用于数据分析。

风险与不适：您在参与本项目中的**所有聊天记录都会被研究者使用**，但这些信息仅用于本研究的数据分析，不会对外披露。同伴支持的微信群聊可能会有一些**不当言论和错误信息**，会对参与者带来**误导或者心理不适**，项目组会在每一个微信群里派一名专业精神科医生，管理和纠正不当言论和信息。

受益：通过参与本项目，您会获得专业而权威的心理健康教育，会获得很多来自同伴的情感和信息支持，同时也会获得精神科医生的一对一在线指导与帮助。您对本项目参与体验的反馈有助于我们对项目进行持续改进，便于后期推广，让更多的患者及家庭受益。

隐私问题：如果您决定参加本项研究，您的**一切个人资料均属保密**。您的档案将保存在有锁的档案柜中，仅供研究人员查阅。您的所有信息以及聊天记录仅供研究者使用于本研究相关的分析。本项研究结果将以**论文或者论著的形式公开报告，**任何报告都**不会披露您的个人信息**。我们将在法律允许的范围内，尽一切努力保护您个人资料的隐私。当您签署了这份知情同意书，代表您同意您的个人信息被用于上述所描述的研究。

如果您因参与这项研究而受到伤害，如隐私被侵犯，您可以获得相应的补偿。

您可以选择不参加本项研究，或者在任何时候通知研究者要求退出研究，您的数据将不纳入研究结果。

您可随时了解与本研究有关的信息资料和研究进展，如果您有与本研究有关的问题，或您在研究过程中发生了任何不适与损伤，或有关于本项研究参加者权益方面的问题，您可以通过电话与我们课题组负责人余钰（+86 13507443533中国；001-203 7459531美国；微信: jiatingbangzhu）联系。

参加者同意声明：

我已经阅读了上述有关本研究的介绍，对参加本研究可能产生的风险和受益充分了解。我是自愿同意参加本文所介绍的临床研究。

签名：_________________________签名日期_________________________
