# Supplementary material for: Assessing a WeChat-Based Integrative Family Intervention (WIFI) for Schizophrenia: Protocol for a Stepped-Wedge Cluster Randomized Trial
Source: JMIR Res Protoc. 2020 Aug 25;9(8):e18538. doi: 10.2196/18538 (PMC7479588; doi:10.2196/18538)
Supplement: Multimedia Appendix 4 [file resprot_v9i8e18538_app4.pdf]

关于国家自然科学基金资助项目批准及有关事项的通知

余钰　　先生/女士：

根据《国家自然科学基金条例》的规定和专家评审意见，国家自然科学基金委员会（以下简称自然科学基金委）决定批准资助您的申请项目。项目批准号：71804197，项目名称：基于“以奖代补”政策下的精神分裂症家庭干预及评估：一项阶梯干预试验研究，直接费用：18.50万元，项目起止年月：2019年01月至2021年12月，有关项目的评审意见及修改意见附后。

请尽早登录科学基金网络信息系统（https://isisn.nsfc.gov.cn），获取《国家自然科学基金资助项目计划书》（以下简称计划书）并按要求填写。对于有修改意见的项目，请按修改意见及时调整计划书相关内容；如对修改意见有异议，须在计划书电子版报送截止日期前提出。

计划书电子版通过科学基金网络信息系统（https://isisn.nsfc.gov.cn）上传，由依托单位审核后提交至自然科学基金委进行审核。审核未通过者，返回修改后再行提交；审核通过者，打印为计划书纸质版（一式两份，双面打印），由依托单位审核并加盖单位公章后报送至自然科学基金委项目材料接收工作组。计划书电子版和纸质版内容应当保证一致。向自然科学基金委提交和报送计划书截止时间节点如下：

1、提交计划书电子版截止时间为**2018年9月11日16点**（视为计划书正式提交时间）；

2、提交计划书电子修改版截止时间为**2018年9月18日16点**；

3、报送计划书纸质版截止时间为**2018年9月26日16点**。

请按照以上规定及时提交计划书电子版，并报送计划书纸质版，未说明理由且逾期不报计划书者，视为自动放弃接受资助。

附件：项目评审意见及修改意见表

国家自然科学基金委员会
管理科学部
2018年8月16日

附件：项目评审意见及修改意见表

<table>
<tr><td>项目批准号</td><td>71804197</td><td>项目负责人</td><td>余钰</td><td>申请代码1</td><td>G040604</td></tr>
<tr><td>项目名称</td><td colspan="5">基于“以奖代补”政策下的精神分裂症家庭干预及评估：一项阶梯干预试验研究</td></tr>
<tr><td>资助类别</td><td colspan="2">青年科学基金项目</td><td>亚类说明</td><td colspan="2"></td></tr>
<tr><td>附注说明</td><td colspan="5"></td></tr>
<tr><td>依托单位</td><td colspan="5">中南大学</td></tr>
<tr><td>直接费用</td><td colspan="2">18.50 万元</td><td>起止年月</td><td colspan="2">2019年01月 至 2021年12月</td></tr>
<tr><td colspan="6">通讯评审意见:
<1>申请人具有创新思维，申请项目立意新颖，以国家政策为导向具有重要的应用前景，研究内容恰当，思路清晰，方案可行。
<2>本研究是在“以奖代补”政策下进行患者家庭干预，家庭干预在精神分裂症的干预中并不少见，但是结合政策背景下的干预是一个最好的研究契机，会带来更好的效果，本研究具有一定的创新性和前沿性，具有研究价值。但研究没有阐述清楚阶梯干预实验与以往的干预实验有哪些特别的优势，建议进一步补充国内外研究文献，以便了解干预的前沿性和可行性。
研究目标和内容较为合理， 研究具体目标可以再细化，以便有的放矢地完成研究。
研究方案较合理，对阶梯干预实验的具体开展步骤，具体内容还需细化。
研究基础较好，经费预算较合理，可以完成研究。

<3>该项目旨在评估该“以奖代补”政策的实际操作情况和效果，并基于此政策，在借鉴国外家庭干预的研究内容和方法的基础上，设计出一个适合中国国情的家庭干预项目对该政策进行进一步的强化，并评估其效果。
总体上，研究目标明确、研究内容适度、关键科学问题选取得当，项目设计有一定创新性。研究紧靠“以奖代补”政策，具有一定的研究价值。总体研究方案基本合理可行。技术路线明确。
该项目作为申请人博士课题的延伸，具有一定的研究基础。另外申请人与研究现场合作关系良好，为研究实施提供了工作条件。
<4>该研究拟采用阶梯干预试验设计对精神分裂症实施基于“以奖代补”政策支持下的家庭干预，从患者，家庭照顾者、家庭、经济方面评估干预效果，为发展精神分裂症家庭管理模式提供科学依据，具有较强的应用价值。该项目负责人及团队成员前期具有较强的研究基础，团队配备合理，立题依据充分，研究内容与理论框架清晰、技术路线合理，研究方法若能补充如何控制阶梯设计中影响结果稳健性的因素并充分阐述统计分析策略与依据，将增强研究方案的科学性。</td></tr>
<tr><td colspan="6">修改意见:

管理科学部

2018年8月16日</td></tr>
</table>
